# Brief considerations on targeting RNA with small molecules

**DOI:** 10.12703/r/11-39

**Published:** 2022-12-21

**Authors:** Quentin Vicens, Eric Westhof

**Affiliations:** 1 Department of Biochemistry and Molecular Genetics, RNA Bioscience Initiative, University of Colorado Anschutz Medical Campus, School of Medicine, Aurora, CO 80045, USA; 2 Université de Strasbourg, Institut de Biologie Moléculaire et Cellulaire, Architecture et Réactivité de l’ARN, CNRS UPR 9002, 2, allée Konrad Roentgen, F-67084 Strasbourg, France

**Keywords:** Drug discovery, RNA structure & dynamics, RNA targeting, SARS-CoV2

## Abstract

For more than three decades, RNA has been known to be a relevant and attractive macromolecule to target but figuring out which RNA should be targeted and how remains challenging. Recent years have seen the confluence of approaches for screening, drug optimization, and target validation that have led to the approval of a few RNA-targeting therapeutics for clinical applications. This focused perspective aims to highlight — but not exhaustively review — key factors accounting for these successes while pointing at crucial aspects worth considering for further breakthroughs.

## Why is it reasonable to target RNA with small molecules?

Most drugs in use today target protein enzymes and receptors^1^. They generally work by controlling or rectifying a biochemical process related to a particular ailment, thereby helping to restore normal functions. Several anti-cancer therapeutics also target DNA^2^. The reason why proteins and DNA have been the predominant target of medicine is historical: their three-dimensional structures^3^ and hence their role in disease were discovered earlier than that of RNA. But we now know that out of all the RNAs that get made in cells during transcription^4,5^, many actually have structure-related regulatory activities whose disruption leads to disease^6^. So, the same idea as for proteins applies to RNA: drugs that bind to RNA could help “correct” faults to support disease remission. RNA just had a later start at being recognized as a suitable drug target for small molecules (aside from being a prime target for antisense oligonucleotides^7^). But as the pool of functional — and hence druggable — RNAs continues to grow, RNA as a therapeutic target is receiving greater attention, as illustrated by an extensive survey published recently^8^.

Like proteins, RNAs may be considered druggable when they possess clefts in which small molecules can bind^9–11^. That said, a ligand becomes a pharmaceutical only when binding to its target leads to a therapeutic effect. Most drugs actually work by competing with an endogenous metabolite^12^ or by targeting an allosteric site^13^. Fortunately, many naturally occurring compounds bind to RNA^14–20^, even though the chemical diversity of the four RNA building blocks is less rich than that of the 20 amino acids that make proteins. For example, metabolites bind to RNA riboswitches in bacteria to influence gene expression, representing potential systems for therapeutic intervention^21^. Furthermore, ribosomal RNA is the prime target of antibiotics like neomycin, streptomycin, and tetracyclin^15,22^. These examples of small molecules binding to RNA in nature indicate that RNA is as well suited as proteins for drug design or screening^23–25^.

## Choosing the right target

Just because RNA is, in principle, a legitimate target for medicine does not mean that *any* RNA can be readily targeted. As for proteins^26^, the process of validating suitable RNA targets can be expected to take many years. Most RNAs exist not as static folds but as ensembles of structurally and temporally dynamic molecules^27–29^. Every atom moves around an average position with possible larger local movements (like pucker changes or base-flipping events), possibly leading to interconversion between structures. To be effective, ligands ought to act by altering the conformational ensemble in a productive manner with favorable binding energetics^27,30^.

**Figure 1.**
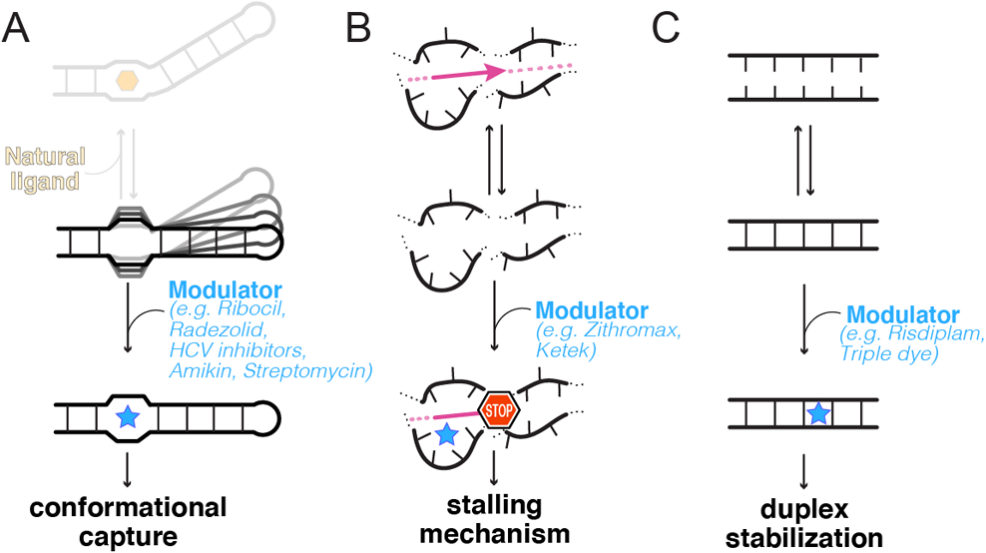
Three ways that modulators of RNA function target molecular dynamics. (**A**) Small molecules select one state for binding out of several, thereby stabilizing a certain fold. The target may have known endogenous ligands (light orange, as in riboswitches) or not (as in the hepatitis C virus [HCV] RNA)^21,45,46^. Drug exemplars are indicated by brand name when available. (**B**) Small molecules modulate the outcome of a biological process catalyzed by RNA (e.g., elongation of a protein chain; pink arrow). Macrolides, for example, affect the translation of some proteins but not others, leading to trapped sequences within the ribosome (pink line with a ‘stop’ sign)^38^. (**C**) Modulators intercalate within RNA duplexes or helical junctions to stabilize a particular intermediate^47,48^.

In that sense, targeting RNA seems most effective when we know about the various interconverting functional RNA structures. Hence, RNAs that have natural cognate ligands have been highly sought after, as endogenous ligands tend to act by altering local molecular transitions or switches between conformations, which can be studied and therefore harnessed for drug discovery. For example, the concentration of metabolites during transcription determines the fate of riboswitches^29,31^, and cofactors are required for the self-splicing of group I ribozymes^32,33^. Natural antibiotics also tend to act by disturbing conformational dynamics: paromomycin and related aminoglycosides interfere with the dynamics of the aminoacyl-tRNA decoding site in the ribosome^34–36^, and streptomycin stabilizes the ribosome in a conformation that supports constant GTPase activation^37^ (Figure 1A). Macrolide antibiotics modulate ribosome function^17^ by allowing the translation of some protein sequences but not others^38^ (Figure 1B). In fact, most efforts in RNA drug targeting to date have focused on developing new drugs that would bind to these well-characterized sites in riboswitches and ribosomal RNA.

The task of developing drugs that interfere with RNA dynamics is much harder when the RNA has no known cognate ligand. It is then problematic to identify which RNA to target, at which location, and which shifts in the conformational ensembles would be associated with a therapeutic outcome. The genomes of RNA viruses were early recognized as attractive targets^23^ because they are littered with functional structured elements that often alternate between states^39^. For instance, studying the human immunodeficiency virus (HIV-1) transactivation response and frameshift stimulation elements led to the identification of many inhibitors^40,41^, some of which were shown to perturb the RNA dynamic ensemble^42^. Similarly, the severe acute respiratory syndrome (SARS) CoV-2 frameshifting element may constitute a suitable target, as this RNA has been proposed to alternate between several structures^43,44^.

But determining which state should be targeted is difficult *a priori* because this requires a deep understanding of the associated functional mechanisms, which takes time to acquire. For example, benzimidazole derivatives were reported to bind *in vitro to* a mimic of domain IIa from the hepatitis C virus (HCV) RNA^49^. Later, it was found that benzimidazoles act at the molecular level by disrupting a conformational switch, as seen in riboswitches^45,50^. In the end, many years later, it was also discovered that these compounds actually compete with a previously unidentified cognate ligand of the HCV RNA^51^.

Do human RNAs constitute suitable targets for small molecules? A naphthyridine derivative was identified that binds to toxic repeat RNA sequences folded as hairpin structures within the human transcriptome, thereby causing shifts in the structural ensemble so that RNA binding proteins can no longer recognize these motifs^52^. It was also recently discovered that the translation readthrough-inducing drug ataluren — used to treat, for example, Duchenne muscular dystrophy — acts by binding to human ribosomal RNA in multiple sites, thereby competing with various factors^53,54^. Finally, the drug risdiplam (marketed as Evrysdi), recently approved by the US Food and Drug Administration (FDA), is a splicing modifier that binds to two locations within a duplex made of two complementary human RNAs without competing with any endogenous ligand. Specifically, risdiplam acts by stabilizing the intermediate complex formed by the pre-mRNA corresponding to the survival of the motor neuron 2 (*SMN2*) gene and the U1 snRNA from the splicing machinery (Figure 1C). As a result, the *SMN2* exon 7 is included, leading to increased production of the functional SMN protein^47,55^. Both ataluren and risdiplam act by modulating conformational states, by precluding recognition by other molecules and by favoring a particular state within the conformational landscape of the RNA. These examples illustrate that the field of RNA drug discovery has reached a stage where drugs can be found that alter the disease outcome by binding to human RNAs. Yet, because discovering suitable targets for either viral or human RNAs remains arduous and largely serendipitous, it is key to continue developing screening approaches^56,57^. These strategies are most effective when they allow the linkage of a desired phenotype to the recognition of a particular RNA target by a small molecule.

## Effective screening

As for protein binders, RNA ligands are often identified through *in silico*, *in vitro*, or *in vivo*^1^ screening — or a combination thereof. But, as it turns out, the tools and strategies developed for protein drug discovery (including molecular dynamics force fields and docking approaches) are not readily applicable to RNA drug discovery. Even when three-dimensional structures are available for the RNA target, the conformation captured, for example, in a crystal structure may not represent the best model to design drugs. *In silico* screening is attractive because of the level of atomic precision it allows, although this is generally still computationally expensive. Molecular dynamics approaches could overcome some of the issues of working with static targets by proposing transient states more relevant for ligand capture by the RNA^58^. Faster and cheaper coarse-grained predictions, though missing atomistic details and resolution, may also offer insights into conformational ensembles^59,60^. However, *in silico* dynamics may not recapitulate *in vivo* transitions, which happen according to precise kinetics and may require partners absent from simulations. Generally, current *in silico* approaches seem most useful on the back end, in combination with *in vitro* and *in vivo* assays, for rationally improving drug efficiency, stability, and bioavailability through structure-activity relationship studies^61–64^.

*In vitro* screening approaches tend to look for a particular biochemical response, such as binding or an increase in expression using a particular reporter system^65^. Many of the currently studied small molecules that bind RNA have been identified by such means. For example, the affinity selection-mass spectrometry approach underlying the automated ligand detection system led to effective modulators of the flavin mononucleotide (FMN) riboswitch^66^ and, more recently, of the Xist long noncoding RNA^67^. Fragment-based approaches have become popular to identify small binders that may eventually be connected to create drugs because they allow an extensive search of the chemical space with limited compound library sizes^68–71^. However, hits from *in vitro* screening may not be clinically pursued if, for example, they elicit no effect or even present off-target effects *in vivo*^72,73^ or if they fail to elude bacterial resistance mechanisms (as seen with ribocil^62^ targeting the FMN riboswitch). Off-target effects, for example, may be spotted early upon counter-screening against such unwanted effects (for examples, see 67,74). *In vitro* screening may also lead to compounds that act by means other than binding RNA. For instance, in the search for small molecule inhibitors of the HCV internal ribosome entry site^45^, hundreds of positive hits from high-throughput screening turned out to act not by binding to the HCV RNA but by inhibiting the luciferase used as a reporter in the assay or by generally interfering with translation^75^. Similarly, although merafloxacin was, for example, identified through a high-throughput reporter screen as a potential SARS-CoV2 RNA inhibitor, whether it acts by directly binding to the frameshifting element remains to be determined^76^.

The development of robust *in vivo* approaches in recent years has been a major leap forward. FDA-approved risdiplam is derived from compounds identified through *in vivo* luminescence-based screening^47,55^. Other compounds, such as branaplam, also a splicing modifier^77^, and ribocil^62,66^, were identified through phenotypic screening as well. Ribocil was isolated from a pool of 57,000 antibacterial small molecules because it inhibited bacterial growth by binding to the FMN riboswitch, an RNA sensor involved in the regulation of genes involved in the riboflavin pathway. Overall, phenotypic screening approaches aim to monitor how a defined function within a particular biological pathway is affected^56^. Such approaches are powerful because the resulting hits were obtained in a natural environment, which may have proteins or other molecules competing with the small molecule that would otherwise be absent from *in vitro* screening. But because phenotypic screening does not solely identify molecules that would bind to a particular RNA, this strategy still benefits from being complemented by biochemical and biophysical assays that will evaluate binding to RNA^8^.

## Upgrading structure-activity relationship strategies

Even when RNA targets have been validated, and effective RNA modulators have been identified, the design of drug leads and their optimizations for increased efficiency and potency brings new challenges. RNA binders benefit from having “RNA-like” physico-chemical characteristics, such as positively charged functional groups, and dipolar as well as hydrophobic/hydrophilic characters, hydrogen bond donors and acceptors, and base-like moieties^78,79^. The drug specificity may be enhanced by the presence of methyl groups or halogen atoms^80–82^. As shown, for example, by drug leads that bind to the FMN riboswitch, productive binding can be achieved equally by various combinations of interaction types: synthetic derivatives of FMN make fewer hydrogen bonds but more van der Waals interactions with the RNA^46,66^. Finding the correct interplay remains challenging^83,84^, especially as changes in the electronic state of the ligand ((de)-protonation or tautomerization) may also occur^85–87^. Meeting such criteria is necessary for binding but not sufficient for selectivity and optimal potency *in vivo*^56^.

**Figure 2.**
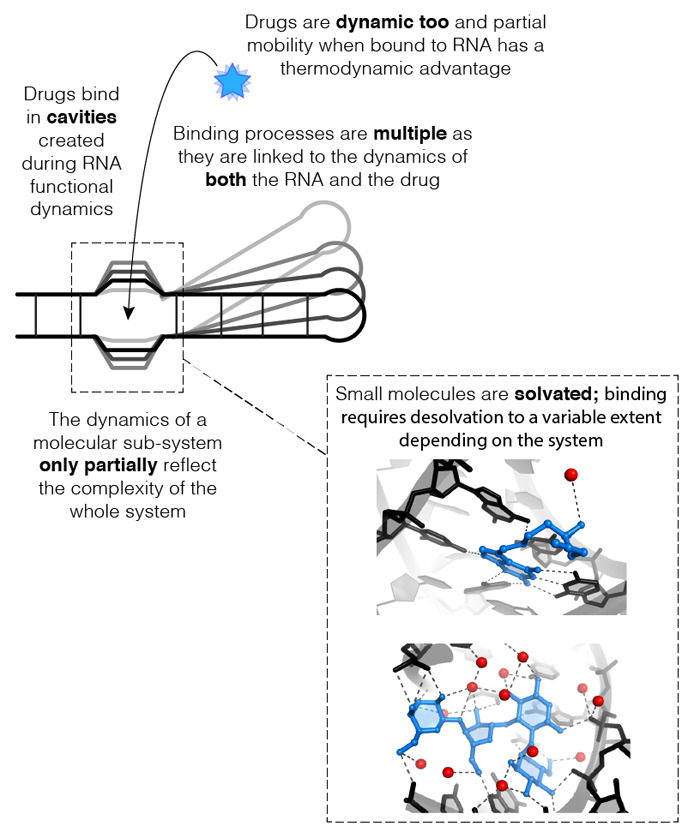
Physico-chemical guidelines for approaching small molecule binding to RNA. The figure highlights how the dynamics of specific and defined regions in RNA folds are critical for biological function. Although the figure uses a similar schematic to Figure 1A, the principles apply to any scenario. Shown in the inset are examples of a mostly desolvated bound ligand (top, pre-queuosine analog 1 bound to the PreQ1 riboswitch; PDB ID 7E9E^88^) and of a drug that retains most of its solvation shell upon binding (bottom, paromomycin bound to ribosomal RNA in a 2.0 Å cryo-electron microscopy [cryo-EM] structure of the bacterial ribosome; PDB ID 7K00^89^). Water molecules are shown as red spheres.

What will likely improve the activity of small molecules is to consider their dynamics: ligands need to slide into cavities by taking advantage of correlated motions in the RNA (Figure 2). Failure to reach the right balance may lead to no effect or even to off-target effects (which basically means that the ligand ends up “preferring to slide” in a target other than the one intended). Such phenomena are complex to anticipate. For example, restricting the two known antibiotics linezolid and sparsomycin by covalently linking them made sense because structural studies revealed that their binding sites on the ribosome overlap^90–92^. The resulting chimeric molecule, now known as radezolid^93,94^ (an antibiotic currently in phase III clinical trials^95,96^), is an illustration of what rational structure-based drug design approaches can achieve. Similarly, covalently linking thiamine and methylene diphosphonic acid increased the affinity to the thiamine phosphate riboswitch by 600-fold over thiamine alone^97^. Yet an equally rational approach aimed to conformationally restrain aminoglycosides in their bioactive conformation did not lead to the expected improvement in activity^98,99^. As it turns out, minimal overall conformational differences in the rigidified antibiotic led to the loss of key interactions with the target^100^, resulting in an even lower binding efficiency than the unconstrained molecule. These examples illustrate the power but also the limitations of our current rational approaches, which should encourage efforts in improving molecular dynamics strategies for drug discovery^58,101^. Cryo-electron microscopy represents a promising avenue for gaining structural along with dynamic information^102^, although when applied to RNA^103–105^, the currently achievable resolutions are not sufficient for structure-activity relationship studies in most cases (except for the ribosome^89^; Figure 2).

Another aspect that may help direct further drug design strategies is to consider the contribution of the solvent. Both the RNA and its ligand are surrounded by ions and water molecules, which constantly exchange on a picosecond timescale. Binding necessitates the partial but complementary and coordinated loss of solvent molecules on both the RNA and the ligand^89,106^. Ligand atoms may take the place of water molecules around the RNA to different extents depending on the compound and its binding mode (Figure 2). Although the role of the solvent was recognized early on^107,108^, it remains largely overlooked in the literature, probably because the contributions of water to stability around a ligand and its target are inherently challenging to fathom. Figuring out which water molecules can be displaced by which functional group to maximize the desired outcome is difficult to predict and measure^109^. Crystal structures of ligand-RNA complexes could help advance the drug optimization process by including solvent considerations, but few such crystals diffract to about 2.5 Å or higher resolution to visualize ordered water molecules. Overall, the free energy of binding results from a combination of enthalpy-driven and entropy-driven contributions, the ratio of which depends on the ligand chemical entities and the geometry and dynamics of the RNA target^25^. Here, biophysical approaches like isothermal calorimetry aimed at characterizing the properties not just of the RNA but also of the ligand and its interactions with the RNA target are key to pinpointing when the physico-chemical properties of both can synergize.

## Looking ahead

Progress with therapeutic interventions aimed at targeting RNA has been slow, whether therapeutic agents are small molecules or antisense oligonucleotides^25^. Every target system displays a particular set of specific molecular characteristics that need to be precisely grasped and measured. Patience, rigor, and humility are key as we progressively refine our understanding of how RNA structures and dynamics support RNA-mediated functions and how they are affected by ligand binding (Figure 2). But the clock is ticking, in particular when antibiotic resistance is now the third leading cause of death in the US^110^. Recent accomplishments in RNA targeting by small molecules can be traced back to structure-based drug design strategies against well-established targets. They were also made possible by phenotypic screening as it bypasses the false positives that typically arise when relying on binding assays only. In addition, such approaches owe their success to realistic expectations about what small molecules can reliably achieve upon binding RNA, such as interfering with a particular conformational changeover. It is likely that future strategies of a similar nature will lead to the discovery of more drugs that regulate RNA-based functions.

## Key points

The dynamics of specific and defined regions in RNA folds are critical for biological function.
Identifying RNA modulators require functional and structural characterization of such regions.
Combining biochemical and phenotypic screening approaches is optimal for identifying specific compounds that may become drugs.
